# Primary Cutaneous Endometriosis of the Umbilicus: A Diagnostic Challenge

**DOI:** 10.7759/cureus.109368

**Published:** 2026-05-21

**Authors:** Xi Na Huang, Farlin Asharaff, Matthew Sommerlad, Soogan Lalla

**Affiliations:** 1 Dermatology, University Hospital Southampton NHS Foundation Trust, Southampton, GBR; 2 Medical Histopathology, University Hospital Southampton NHS Foundation Trust, Southampton, GBR

**Keywords:** cutaneous endometriosis, cyclical umbilical nodule, endometrioma, extrapelvic endometriosis, immunohistochemistry, umbilical endometriosis, umbilical nodule, villar's nodule

## Abstract

Umbilical endometriosis, or Villar’s nodule, is a rare form of cutaneous endometriosis in which ectopic endometrial tissue involves the umbilical skin. It may occur without any history of abdominal or gynaecological surgery, making diagnosis difficult and leading to misdiagnosis as more common umbilical lesions.

We describe a 31-year-old woman with a 2-year history of an enlarging umbilical lesion associated with intermittent swelling, discomfort, and persistent moisture, with flares around menstruation. She was treated repeatedly in primary care for presumed infection or umbilical granuloma using topical antifungals and antibiotics, without sustained improvement. Examination revealed a polypoid, lobulated violaceous-brown nodule within the umbilicus. Ultrasound demonstrated a vascular pedunculated nodule confined to the anterior abdominal wall. Punch biopsy showed endometrial-type glands and stroma in the dermis, and immunohistochemistry confirmed cutaneous endometriosis.

She was referred to gynaecology for evaluation of possible pelvic endometriosis and to plastic surgery to consider surgical excision and umbilical reconstruction; definitive surgery is pending at the time of writing. This case highlights umbilical endometriosis as an important differential diagnosis in women presenting with umbilical nodules and cyclical symptoms and underscores the role of histopathology in distinguishing Villar’s nodule from malignant or metastatic umbilical lesions.

## Introduction

Umbilical endometriosis, also known as Villar’s nodule, is a rare form of cutaneous endometriosis in which endometrial glands and stroma are found in the umbilical skin [1,2]. It represents a very small proportion of all extrapelvic endometriosis and may occur without previous abdominal or gynaecological surgery, which can delay recognition [2,3].

Umbilical endometriosis accounts for an estimated 0.5-1% of extrapelvic endometriosis and may be primary (spontaneous) or secondary (occurring in scars following laparoscopic or obstetric/gynaecological surgery) [2-4]. Several mechanisms have been proposed, including lymphatic or haematogenous spread of endometrial cells, retrograde menstruation with peritoneal implantation, and metaplastic change of local tissue [2,5,6].

Clinically, patients often report a painful or tender umbilical nodule that may change in size, colour or consistency during menstruation; bleeding or discharge can also occur [2-4]. These features can mimic more common umbilical conditions, contributing to misdiagnosis and delayed recognition. The main differential diagnoses include umbilical granuloma, pyogenic granuloma, epidermoid cyst, dermatofibroma, keloid, primary or metastatic cutaneous malignancy (including Sister Mary Joseph nodule) and umbilical hernia [2,4].

## Case presentation

**Figure 1:**
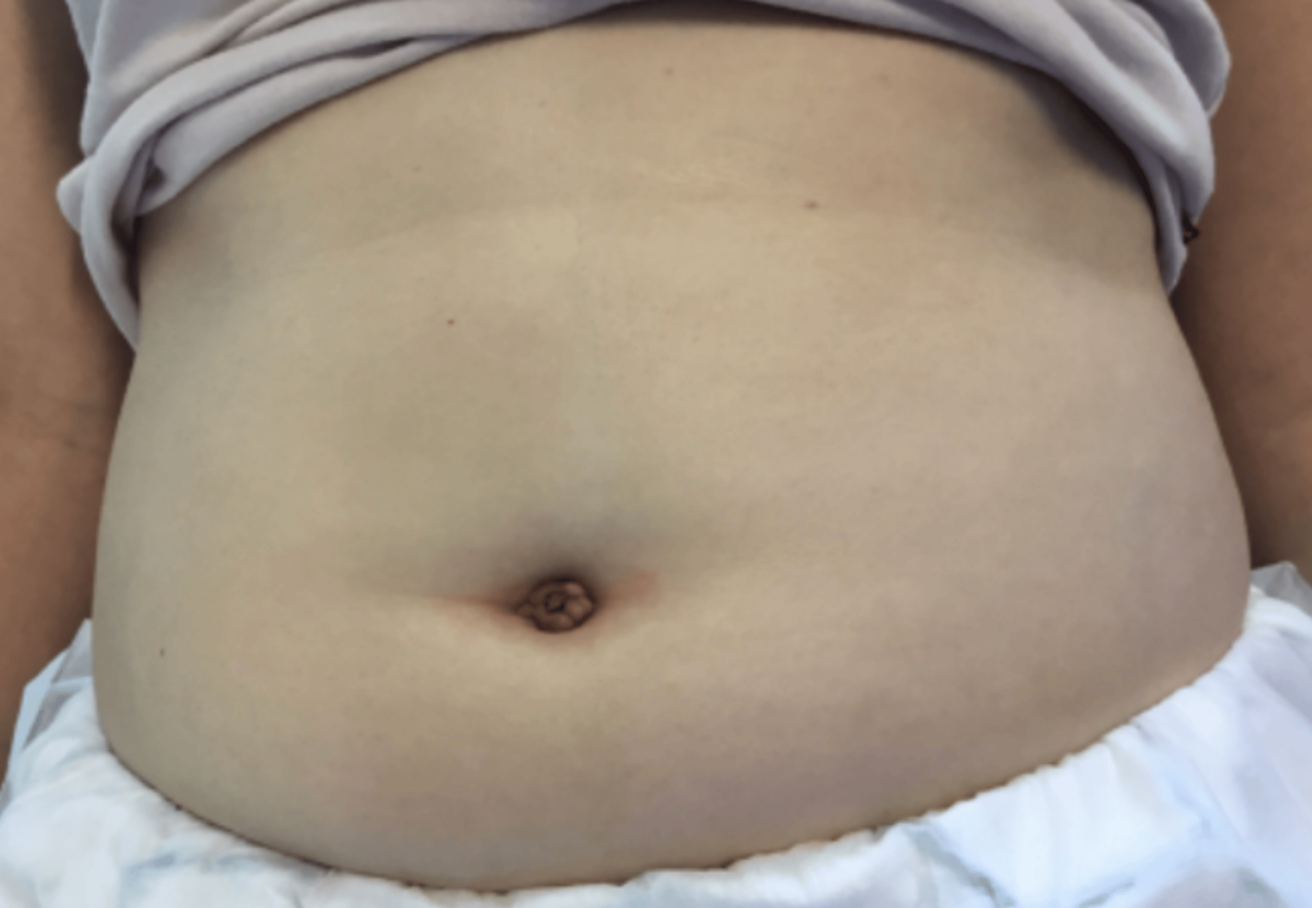
Anterior abdominal view: solitary protruding umbilical lesion with no other abdominal wall abnormality

A 31-year-old woman presented to the dermatology clinic with a 2-year history of an enlarging umbilical lesion associated with intermittent swelling, discomfort, and persistent moisture (Figure 1). Two episodes of increased tenderness over the past year occurred around the time of her menstrual periods. She had been treated several times in primary care for presumed umbilical infection or granuloma with topical antifungals and antibiotics, without sustained improvement. Her medical history included a right ovarian endometrioma (“chocolate cyst”) diagnosed on ultrasound in 2018-2020 and longstanding dysmenorrhoea. She had never undergone abdominal or pelvic surgery. She was nulliparous, otherwise well, and took no regular medications. On examination, there was a solitary, approximately 15 mm polypoid nodule arising from within the umbilicus. The lesion was lobulated and violaceous-brown, with a short pedunculated stalk and mild surrounding erythema. There was no clinical evidence of hernia or other abdominal wall abnormality.

**Figure 2:**
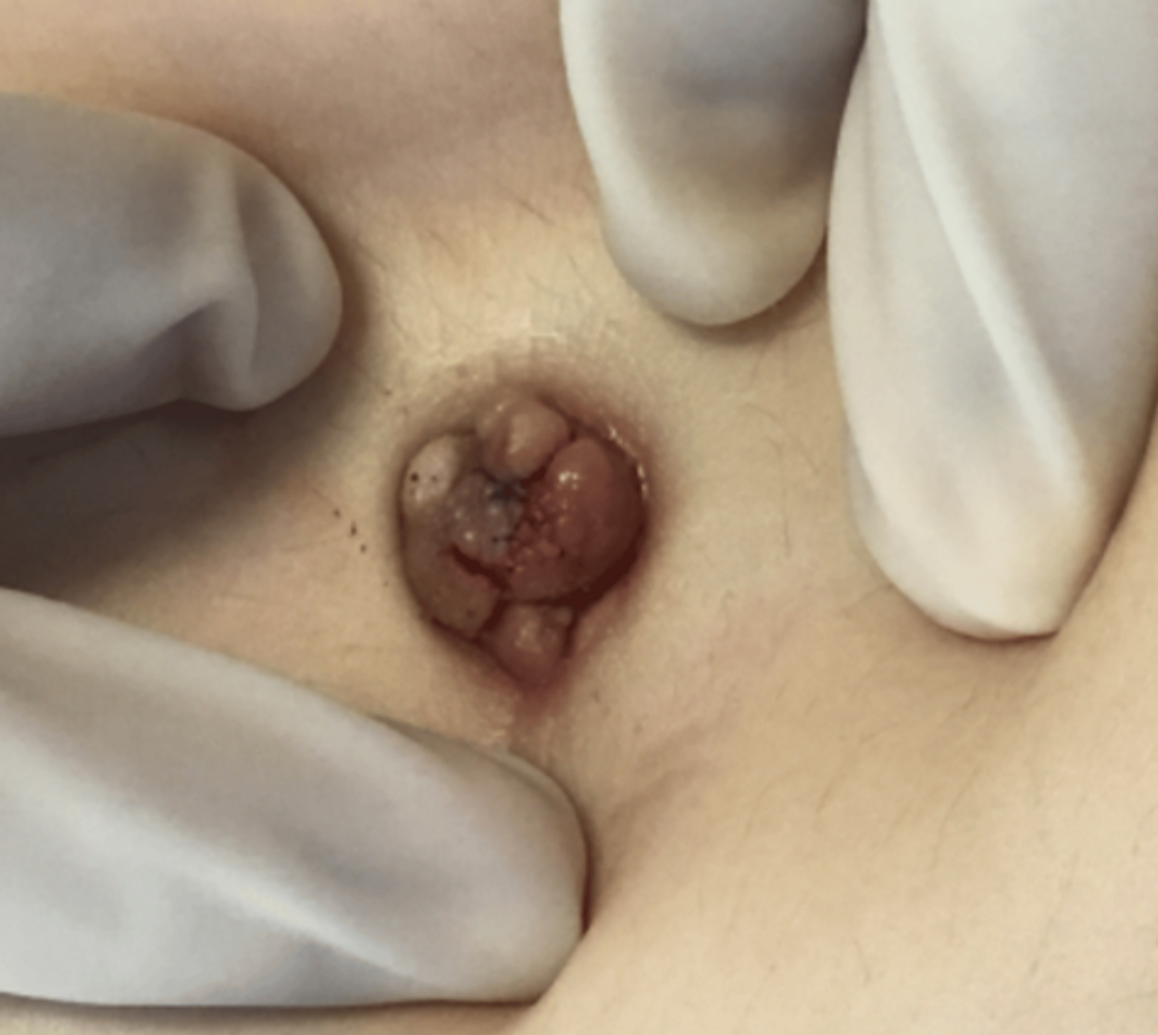
Close-up: polypoid, lobulated, violaceous-brown nodule protruding from the umbilicus with mild surrounding erythema

**Figure 3:**
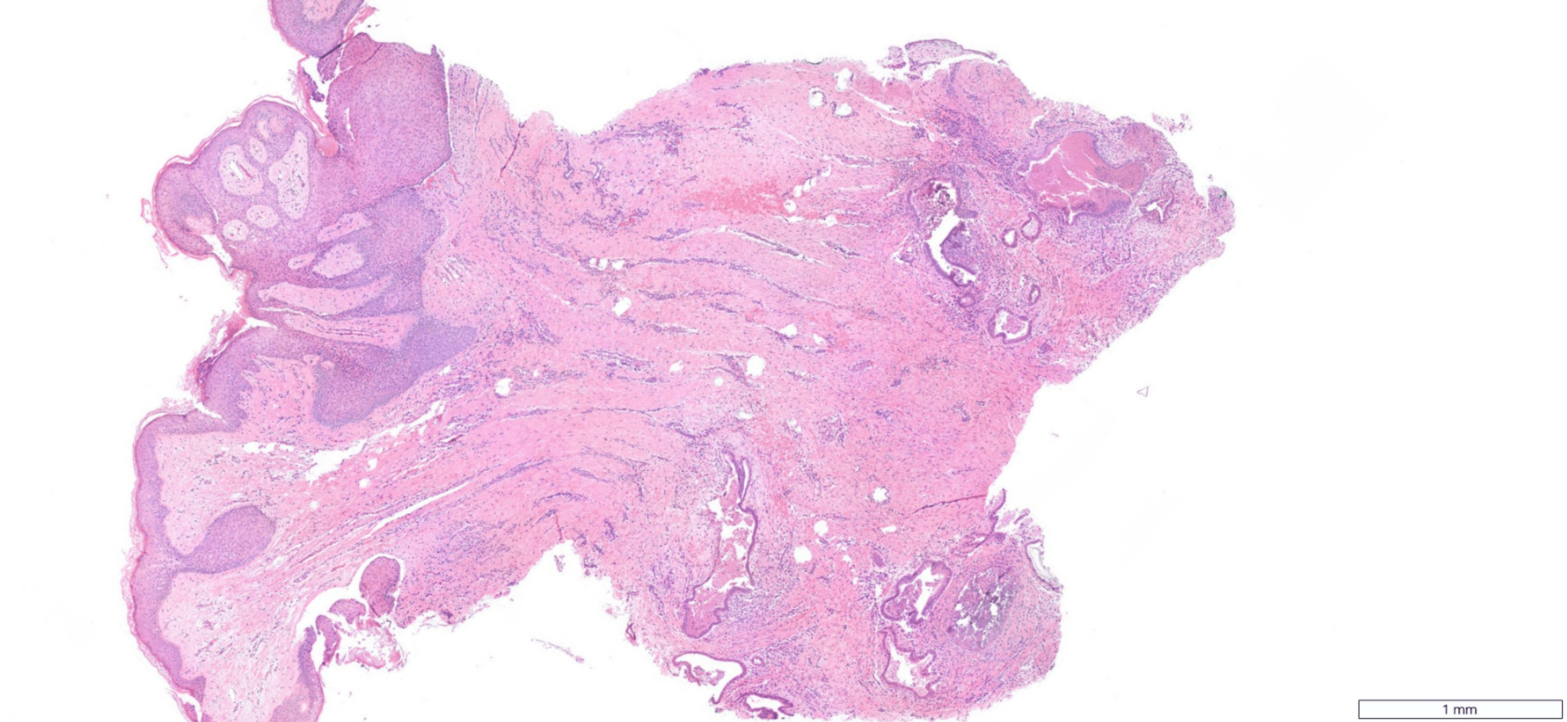
Punch biopsy, H&E, low power (scale bar 1 mm) Dermal fibrosis and chronic inflammation with irregular endometrial-type glands surrounded by a cellular stroma at the deep aspect, in keeping with endometriosis

**Figure 4:**
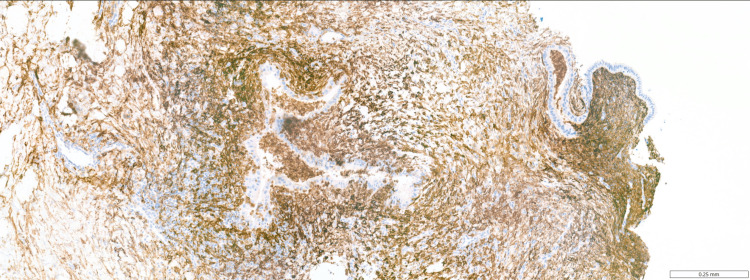
CD10 immunohistochemistry, high power (scale bar 0.25 mm) Positive expression in the stromal cells surrounding the glands, confirming a Müllerian stromal component

**Figure 5:**
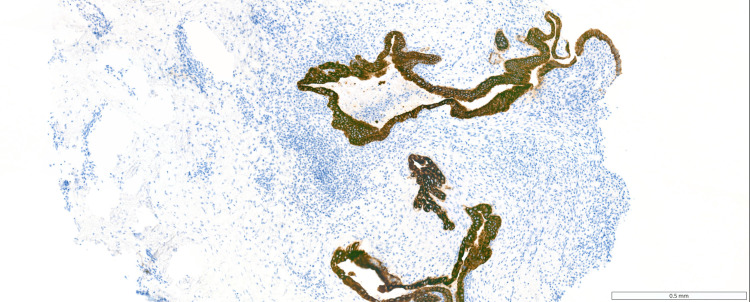
CK7 immunohistochemistry, high power (scale bar 0.5 mm) Positive CK7 expression in the glandular epithelium, in keeping with endometriosis

**Figure 6:**
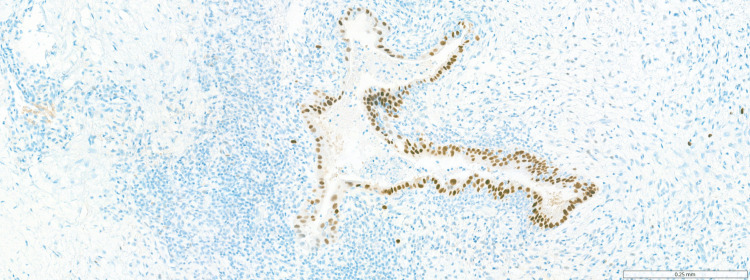
Oestrogen receptor (ER) immunohistochemistry, high power (scale bar 0.25 mm) Positive nuclear expression in the glandular epithelium, supporting a Müllerian origin

Ultrasound of the anterior abdominal wall demonstrated a soft-tissue nodule measuring 14 mm in maximum diameter on imaging (clinically estimated at approximately 15 mm) related to the umbilicus (Figure 2). It was of homogeneously low echogenicity, showed internal vascularity on Doppler and appeared pedunculated, arising from deeper tissue on a thin stalk. There was no associated fluid collection, hernia or communication with the intra-abdominal cavity, in keeping with a superficial soft-tissue lesion. A 4 mm punch biopsy of the nodule was performed. Histology showed skin with fibrosis and chronic inflammation in the dermis, and at the deep aspect, irregular glands were surrounded by a cellular stroma, as shown in Figure 3. Immunohistochemistry demonstrated CD10 positivity in stromal cells (Figure 4), and oestrogen receptor and CK7 positivity in the glandular epithelium (Figures 5-6, respectively), consistent with cutaneous endometriosis [5].

The patient has been referred to gynaecology for evaluation of possible pelvic endometriosis and to plastic surgery for consideration of surgical excision and potential umbilical reconstruction. Definitive surgery is pending at the time of writing. A gynaecology assessment for coexisting pelvic endometriosis has been arranged, and the patient remains under follow-up. Outcome data, including operative and histopathological findings and post-operative course, will be reported once definitive management is complete.

## Discussion

Endometriosis is defined by ectopic endometrial glands and stroma, most commonly within the pelvis, but it has been reported in many sites, including the abdominal wall and umbilicus [1,2]. Only a small number of cases of primary umbilical endometriosis have been reported in the literature, reflecting the rarity of this condition [3,6-8]. Clinically, patients often report a painful or tender umbilical nodule that may change in size, colour or consistency during menstruation; bleeding or discharge can also occur [2-4]. Some series suggest that around 15% of patients with primary umbilical endometriosis have coexisting pelvic endometriosis, highlighting the importance of gynaecological assessment once the diagnosis is established [3]. Imaging is useful to define the extent of the lesion and to exclude hernia, urachal anomalies or deeper masses, although no modality is entirely specific [1,4,7]. In this case, ultrasound demonstrated a vascular polypoid nodule without intra-abdominal extension, which supported a benign superficial process. Definitive diagnosis relies on histopathology, with demonstration of endometrial-type glands and stroma, often accompanied by haemorrhage and chronic inflammation [1,5].

Immunohistochemistry, such as CD10 expression in stromal cells and ER/PR or CK7 expression in glandular epithelium, supports a Müllerian origin and helps distinguish endometriosis from metastatic adenocarcinoma or adnexal tumours [1,5]. Wide local excision is regarded as the treatment of choice for umbilical endometriosis, as it provides tissue for diagnosis, achieves symptom control and minimises recurrence and the small risk of malignant transformation [1,4,6]. Reported recurrence rates after surgical resection range from approximately 5.4% to 27%, but extended excision, including the peritoneum with umbilical reconstruction, has been associated with substantially lower recurrence rates [6]. Hormonal therapy (e.g. combined oral contraceptives, progestins or GnRH analogues) may improve symptoms but appears less reliable as a sole treatment for cutaneous lesions [4,6]. Given the reported association with pelvic endometriosis, gynaecological evaluation and, where appropriate, laparoscopic assessment are recommended to identify additional foci of disease [2,3,8].

## Conclusions

Umbilical endometriosis (Villar’s nodule) should be considered in women presenting with umbilical nodules and cyclical symptoms. Ultrasound can help characterise the lesion and exclude hernia or urachal pathology, but histology with immunohistochemistry confirms the diagnosis and helps distinguish this from malignant or metastatic umbilical lesions. Wide local excision is the preferred treatment and reduces the risk of recurrence or malignant transformation. Identification of cutaneous endometriosis should prompt assessment for coexisting pelvic disease.
